# Madden–Julian Oscillation prediction skill of a new-generation global model demonstrated using a supercomputer

**DOI:** 10.1038/ncomms4769

**Published:** 2014-05-06

**Authors:** Tomoki Miyakawa, Masaki Satoh, Hiroaki Miura, Hirofumi Tomita, Hisashi Yashiro, Akira T. Noda, Yohei Yamada, Chihiro Kodama, Masahide Kimoto, Kunio Yoneyama

**Affiliations:** 1 Japan Agency for Marine-Earth Science and Technology, Yokosuka 237-0061, Japan; 2 Atmosphere and Ocean Research Institute, The University of Tokyo, Kashiwa 277-8568, Japan; 3 Department of Earth and Planetary Science, The University of Tokyo, Tokyo 113-0033, Japan; 4 Advanced Institute for Computational Science, RIKEN, Kobe 650-0047, Japan

## Abstract

Global cloud/cloud system-resolving models are perceived to perform well in the prediction of the Madden–Julian Oscillation (MJO), a huge eastward -propagating atmospheric pulse that dominates intraseasonal variation of the tropics and affects the entire globe. However, owing to model complexity, detailed analysis is limited by computational power. Here we carry out a simulation series using a recently developed supercomputer, which enables the statistical evaluation of the MJO prediction skill of a costly new-generation model in a manner similar to operational forecast models. We estimate the current MJO predictability of the model as 27 days by conducting simulations including all winter MJO cases identified during 2003–2012. The simulated precipitation patterns associated with different MJO phases compare well with observations. An MJO case captured in a recent intensive observation is also well reproduced. Our results reveal that the global cloud-resolving approach is effective in understanding the MJO and in providing month-long tropical forecasts.

## Abstract (web summary)

Prediction of the Madden–Julian Oscillation using complex cloud-resolving models has been limited by computational power. Here, Miyakawa *et al.* run a series of simulations using the newly developed 10 peta-flop ‘K computer’ and demonstrate a Madden–Julian Oscillation predictive window of 27 days.

## Graphical abstract

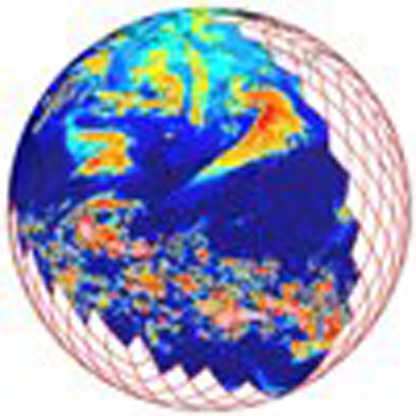

The Madden–Julian Oscillation^1^,^2^ (MJO) is an eastward propagating atmospheric pulse with a typical lifespan of 30–60 days, most clearly visualized in the tropical Indian and Pacific Oceans as a massive envelope that consists of clouds of various horizontal scales. MJO events affect the tropics with heavy rainfall, produce conditions favourable for tropical cyclone developments^3^ and induce Rossby-wave trains that cause sustained anomalous conditions in the extra tropics^4^, for example, heat/cold waves. Owing to its elusive mechanism and uncertainty in behaviour of unresolved clouds represented by simplified approximation schemes called cumulus parameterization^5^ (CP), representation of the MJO remains problematic in many general circulation models (GCMs)^6^. While CP is regarded as a core issue in improving MJOs in conventional GCMs, the Nonhydrostatic Icosahedral Atmospheric Model^7^ (NICAM), a new-generation model developed under the concept of ‘explicitly resolving clouds’, has successfully reproduced an MJO convective envelope and its eastward migration^8^ avoiding the use of CP. However, the large amount of required computational resource has limited such experiments to a small number of case studies. The MJO prediction skill of the model has been perceived to be high but was unable to be assessed. The K computer^9^, a recently developed 10 peta-flops supercomputer broke the computational barrier. We conduct a series of 40-day MJO simulations on the K computer using NICAM to estimate its current MJO predictability. Details of the simulation members and the configuration of NICAM are outlined in Methods. The K computer won the first place on supercomputer ranking by TOP500 ( http://www.top500.org/) in 2011. It has been widely accessible to researchers and companies for scientific and industrial use since September 2012, and is the most powerful computer currently available to the meteorological science community. The finest global mesh of NICAM achievable on the K computer is currently 870 m (ref. 10). Month-long global simulations at 3.5-km mesh are now available, compared with only a single 1-week simulation^8^ completed on the Earth simulator, which ranked highest in 2004.

In this study we use the enhanced computational power of the K computer to increase the number of simulations to enable statistical evaluation instead of applying high resolutions for small numbers of simulations. We apply a 14 km mesh that marginally resolves^11^ meso-beta-scale (20–200 km) convective clouds (hereafter referred to as cloud systems). Meshes of 12–14 km have produced eastward propagation of the MJO convective envelope successfully in previous studies by regional and global models without CP^12^,^13^. We show from 54 simulations that NICAM has the potential to provide a valid prediction of MJO phase for 27 days, and that the precipitation anomaly structures associated with different MJO phases compare well with observation. We also show that NICAM well reproduces an MJO case that was captured in a recent intensive observation project. The high-quality observation and simulation provides together an opportunity for detailed mechanism studies. Our results reveal that the ‘global cloud-resolving’ approach is effective in understanding the MJO and providing month-long tropical forecasts.

## Results

### MJO skill score

**Figure 1:**
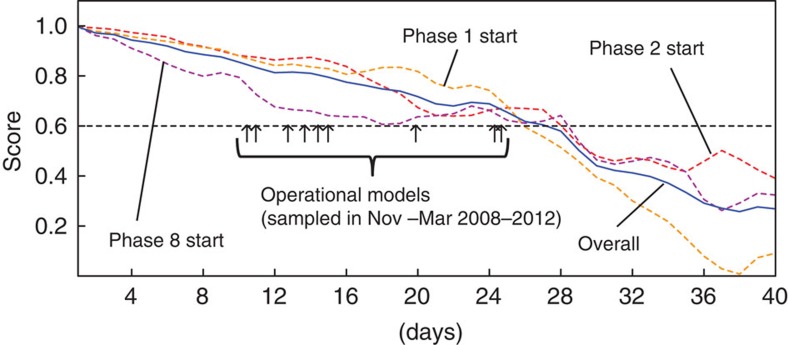
The Madden–Julian Oscillation (MJO) skill scores of Nonhydrostatic Icosahedral Atmospheric Model (NICAM) plotted along the lead time from initial dates. Blue solid plot shows the overall skill score (bivariate correlation; COR) for all 54 simulations. Broken plots show COR for groups of simulations initialized at phase 8 (purple, 17 members), phase 1 (orange, 18 members) and phase 2 (red, 19 members). Arrows indicate the durations COR>0.6 is maintained by recent operational models^14^.

Figure 1 shows the MJO skill score of NICAM plotted against the lead times from the initial dates, along with prediction limits of recent operational forecast GCMs^14^. The MJO skill score is defined by the bivariate correlation^15^,^16^ (COR) between the observed and simulated real-time multivariate MJO (RMM) indices^17^. Details of RMM, COR and procedures for MJO case identification and initial date assignment are described in Methods. NICAM maintains COR higher than 0.6 for 26–28 days depending on the initial MJO phase, 27 days when all 54 cases are included. Phases 8, 1 and 2, respectively, correspond to when the centre of MJO convection is located over South America, Atlantic Ocean—Africa and the western Indian Ocean (Supplementary Fig. 1). GCMs that maintain COR higher than 0.5–0.6 for more than 2 weeks are typically the better models in terms of the MJO predictions^14^,^18^. The Integrated Forecast System model developed by the European Centre for Medium-Range Weather Forecasts is arguably the current best performing GCM in terms of MJO prediction; the newest version maintaining COR higher than 0.6 for 26 days^19^. It is important to note that Fig. 1 is not a comparison between completely equivalent samples from NICAM and operational models due to data availability. The major differences are the following: (a) skill scores of operational models are calculated for simulations initialized at all days in winter, whereas the simulation series of NICAM only include limited initial dates that belong to phase 8, 1 or 2 of either already significant or developing MJOs, and (b) only the operational models apply ensemble forecasts^16^ (the sample differences are further described in Methods). However, the result reveals that the MJO prediction skill of the developing new-generation model is already competitive with that of the top performing, carefully adjusted operational GCMs.

### Precipitation anomaly structures associated with MJO phases

**Figure 2:**
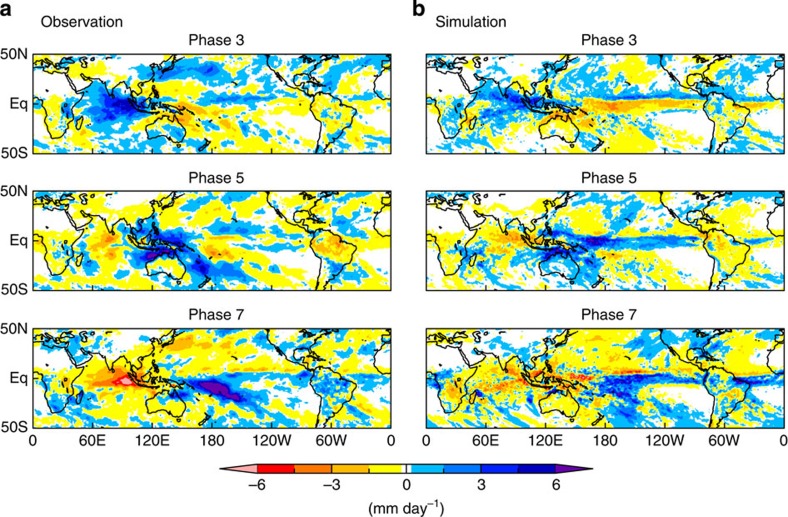
Precipitation anomaly composites for different Madden–Julian Oscillation (MJO) phases. (**a**) One-Degree Daily product version 1.2 by the Global Precipitation Climatology Project^20^, and (**b**) Nonhydrostatic Icosahedral Atmospheric Model (NICAM). Average days from the initial dates of the simulations are 16 days (phase 3), 25 days (phase 5) and 28 days (phase 7). Resolution of the simulation output is lowered to 1° mesh to equal the resolution of the observational data set. Anomalies are calculated as deviations from 40-day mean values of each case.

Although COR is useful for evaluating prediction skills in terms of MJO phases, it disregards MJO structures. In particular, rainfall anomaly patterns that accompany MJO signals deserve validation, considering their large impact on human society. Figure 2 compares composites of observed^20^ and simulated precipitation anomalies for MJO phases 3, 5 and 7. Horizontal structures of the simulated precipitation anomalies well resemble the observations, even for phase 7, for which the average lead time is 28 days. An overestimation of signal in the Inter-Tropical Convergence Zone over the central Pacific found in phase 5 is a bias frequently caused by NICAM^12^. It is probably the result of under-resolved local vertical moisture transport, which could be a common problem for models that explicitly calculate cloud systems without CP at this resolution. More diagnostics are provided in Supplementary Figs 2–9 (composited RMM diagrams, lagged composites of phase probability distribution function, lagged composites and phase-composited vertical structures of MJO-related quantities).

### CINDY2011/DYNAMO case

The simulation series includes an MJO case that occurred in November–December 2011 during the CINDY2011/DYNAMO field campaign^21^,^22^, arguably the most intensive international MJO observation project ever. Recent studies^23^,^24^ have explored model prediction skills for this case in detail, and the Vertical Structure and Diabatic Process of the MJO^25^,^26^ selected the case for their next cross-institutional model inter-comparison. The number of simulations of this particular case in our series is too small for statistical skill evaluations. However, if simulations by NICAM are successful, which appears to be the case as shown below, it will reinforce such activities by providing useful information that enable detailed mechanism studies. It will also provide the opportunity to compare a global cloud system-resolving simulation with a major *in situ* observation for the same MJO case for the first time.

**Figure 3:**
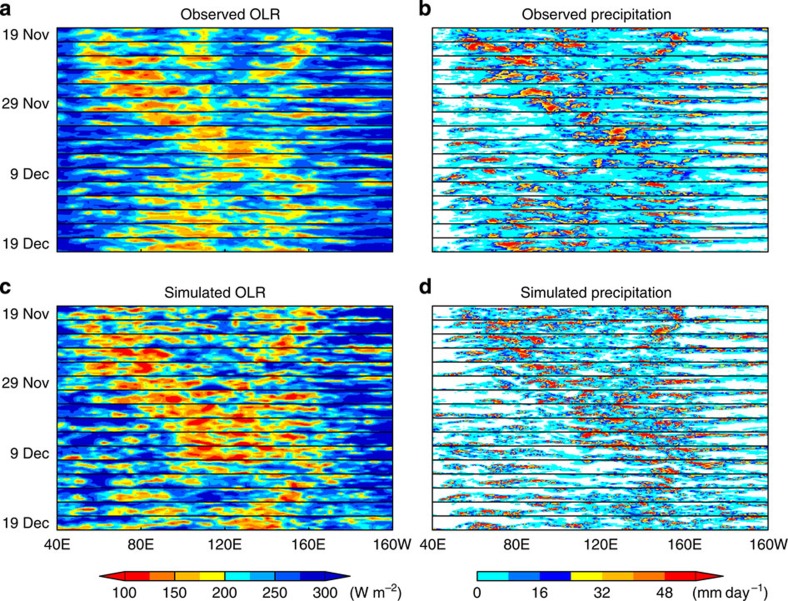
Two-daily series of cloud and rain over the tropical Indian to western Pacific Ocean. (**a**) Interpolated outgoing longwave radiation (OLR) by the National Oceanic and Atmospheric Administration polar-orbiting series of satellites^27^ (NOAA-OLR) and (**c**) OLR by a simulation initialized at 00 UTC 17 November 2011. Smaller OLR values correspond to higher cloud tops. (**b**) Precipitation by the 3B42 product of the Tropical Rainfall Measuring Mission (TRMM) satellite^28^ and (**d**) precipitation by the simulation. The figures consist of slices that show horizontal snapshots of the tropical Indian to the western Pacific Ocean (10S–10N, 40E–160W, indicated in Supplementary Fig. 10). The resolution of the simulated OLR is lowered to 2.5° mesh to equal the resolution of the NOAA-OLR data set. The resolutions of the TRMM 3B42 data set and the simulated precipitation are lowered to 1° mesh.

Figure 3 shows two-daily snapshot series of outgoing longwave radiation^27^ (OLR) and precipitation^28^ over the tropical Indian to western Pacific Ocean (10N–10S, 40E–160W; Supplementary Fig. 10), from 19 November to 19 December 2011. The simulation is a single run initialized at 00 UTC 17 November 2011. A convectively active region of the MJO forms at 50E–90E and migrates eastward from the Indian Ocean to the western Pacific, both in observation and simulation. The leading edge of the simulated convective envelope travels eastward at ~5° per day, a speed similar with the observation. The contrast of OLR between the convective regions and the surrounding regions is larger in the simulation than the observation, possibly because isolated sub-10 km scale clouds in the surrounding convectively suppressed regions are not permitted in the 14-km mesh. Such contrast is reduced in a similar simulation applying a 7-km mesh (Supplementary Fig. 11).

**Figure 4:**
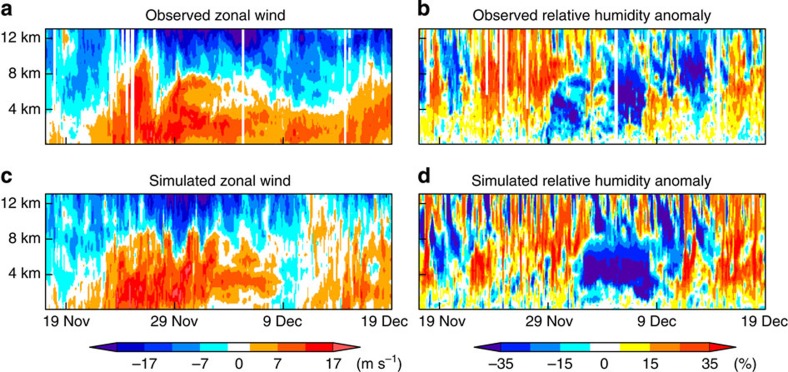
Time–height cross-sections of zonal wind and relative humidity anomalies. Panels **a** and **b** are time–height cross-sections of zonal wind and relative humidity anomalies observed at Gan Island (73.2E, 0.7S, indicated in Supplementary Fig. 10) during the CINDY2011/DYNAMO field campaign^21^. Panels **c** and **d** are time–height cross-sections of zonal wind and relative humidity anomalies from the corresponding grid point in the simulation.

Figure 4a,c shows time–height sections of observed and simulated zonal wind over Gan Island (73.2E, 0.7S; Supplementary Fig. 10) for the MJO case shown in Fig. 3. The model captures the timing of westerly wind intrusion observed on 21 November. The simulated depth of the westerly wind is about 8 km, which is consistent with the observation. Despite a lead time of nearly 4 weeks, the deepening of the westerly wind accompanied by the evolution of the next MJO-like signal is also captured near 15 December. Figure 4b,d shows similar sections for relative humidity anomalies. Water vapour in the lower-middle troposphere has strong control on the depth and organization of tropical convections, and is presumed to be a key factor for MJO evolution^21^. The model captures the transition of moisture condition in the lower-middle troposphere (about 3–8 km), that is, the moistening that accompanies the first MJO, the dry phase that follows and the re-moistening of the second signal. While it is not surprising that the model misses the lower tropospheric westerly wind around 9 December, considering the 3-week lead time, it is interesting that the model still develops the second signal. It may be an indication that neither westerly moisture advection nor local enhancement of wind-induced surface evaporation around 9 December played vital roles in setting up convectively favourable conditions for the second signal.

## Discussion

The physical reason why resolving cloud systems improve the MJO simulations remains an open question. It is noteworthy that the model performs well despite the lack of fine structures of cloud systems in the initial data, which is a simple interpolation from a lower-resolution data set. Thus, subtle details of individual cloud systems are unlikely to be the sources of high predictability. Statistical behaviours of feedbacks from cloud systems in realistic relation with large-scale conditions are the key issues, improved here by explicit calculation of the cloud systems. Finer representation of generation/diffusion and redistribution of heat, mass and momentum by the cloud systems are possible contributors. A recent study^29^ based on an analysis of an MJO case simulated by NICAM discusses that the convective momentum transport, a crudely represented component in current GCMs, may play a role in altering the eastward speed of the MJO, and/or the tilt of its vertical structure, and in enhancement of surface evaporation. Detailed cloud microphysics (for example, condensation/evaporation, freezing/melting, distinction of ice/snow/graupel, aggregation and so on) is also a possible contributor, which may lead to an improved representation of the balance between convective heating and cloud-radiative cooling of the MJO.

Achievement of high-quality global sub-kilometre simulations—which we may call global cloud-resolving simulations—is regarded as a key step needed for a quantum leap in the field, as emphasized by the leaders of climate and meteorological science in a 2008 summit^30^,^31^. Computational powers have improved since then, with the top four supercomputers now sitting above the 10 peta-flops plateau. Our results show that the increase of computational power has efficiently pushed global cloud-resolving modelling activity forward, carrying it to a higher stage where simulations executed at resolutions that marginally resolve meso-beta-scale cloud systems can be statistically assessed, compared with operational forecasts, and be seriously considered for social use. More variations of global cloud/cloud system-resolving models are being developed, for example, MPAS^32^, ICON^33^. The high performance of NICAM is a promising indication that these models have the potential to take over as the new standard in the near future, thanks to the seemingly never-ending innovation of supercomputers. The increase of computational resource will open the scope for ensemble forecasts^16^ by these models, that are likely to further improve prediction skills, and provide additional information on uncertainties of the predictions. Conventional GCMs with CP remain important, however, especially for long-term future climate projections. Global cloud/cloud system-resolving simulations will contribute to improvements of CP by providing quantitative information on condensation/evaporation and on convective transport of mass, heat and momentum. Detailed comparisons with models that apply CP, such as in Project Athena^34^,^35^ that compared previous versions of NICAM and Integrated Forecast System, are essential in distinguishing the effects of cloud/cloud system features that need to be included in models for skilful MJO prediction. Hybrid models that substitute CP with two-dimensional cloud-resolving models called ‘super-parameterization’ also deserve detailed comparison^5^. The new paradigm of global cloud/cloud system-resolving models mark the arrival of a month-long MJO prediction era, play key roles in the improvement of climate simulations and promote mechanism studies of the MJO.

## Methods

### Model configuration

We use the 2012 summer version of NICAM (NICAM.12) modified for the K computer. Governing equations are fully compressible and non-hydrostatic. Finite volume method is applied for spatial discretization. Icosahedral grid system modified by spring dynamic smoothing is applied for horizontal grids^36^,^37^. The model has a terrain following grid system with 38 vertical layers. The model top height is 38 km, and layer thicknesses gradually increase with altitude. The model is run without CP. Applied physics schemes are the NICAM Single-Moment Water 6 cloud microphysics scheme^38^, which considers six categories of hydrometers as prognostic variables, a modified version of the Mellor–Yamada turbulence scheme^39^,^40^,^41^, two-stream radiative transfer with a correlated *k*-distribution scheme MSTRNX^42^, which is an improved version of Louis scheme^43^,^44^ for land surface fluxes, and the MATSIRO land surface model^45^. A mixed-layer ocean^46^ model is used, that is, surface heat flux affects the sea surface temperature (SST) as well as the atmosphere. The ocean mixed-layer is 15 m in depth, and its temperature is nudged to externally provided SST at an e-folding time of 7 days. Initial conditions of the atmosphere and the ocean are derived by linear interpolation of the European Centre for Medium-Range Weather Forecasts Reanalysis Interim (ERA-Interim)^47^, without any additional initialization process. The external SST is defined as the sum of the time-varying mean annual cycle and constant anomalous component. The difference from the mean annual cycle is averaged over the week before the initial date to derive the anomalous component. Thus, no information observed after the initial date is used in the simulations. The simulations are conventionally called ‘hindcast simulations,’ meaning that they take place after the actual occurrence in the real world. By prohibiting use of any data observed after the initial dates, we imitate ‘forecasts’ in this hindcast simulation series.

### The RMM index method

The RMM index method is a broadly accepted technique for monitoring the MJO phase and amplitude. The RMM indices are derived from equatorial zonal winds at 200 hPa, 850 hPa and OLR that account for cloud activities. The eastward migration of the MJO projects on to a two-dimensional phase space spanned by two principle components (RMM1 and RMM2) as a counterclockwise succession of plots (Supplementary Fig. 1). The phase space is conventionally divided into eight phases, each corresponding to a different longitudinal region. The procedures used in this study to objectively identify MJO cases, determine the initial dates and evaluate MJO prediction skills are based on the RMM index method.

### MJO case identification and initial date assignment

We limit our focus to 2003–2012, a period during which observational data assimilated into the ERA-Interim reanalysis are abundant^47^. We focus on boreal winter (October–March), the season during which MJO signals are more clearly distinguished from the Asian monsoon circulation. We identify target MJO cases when a succession of observational RMM plots move counterclockwise through phase 2 to phase 5 (corresponds to eastward propagation of the MJO pulse through the Indian Ocean to the western Pacific) without retreating more than one phase, and have an average amplitude greater than 1 over the span. Nineteen cases meet the criteria. For each MJO case extracted, the first day the RMM plot falls in phase 2 is assigned as the initial date of a 40-day simulation. If it is possible to track the RMM diagram back to phase 1 (phase 8), the first day the RMM plot falls in phase 1 (phase 8) is also assigned as an initial date. Of the 19 MJO cases extracted, 17 were traced back to phase 8. One case was only traceable to phase 1, and another case was not traceable beyond phase 2. Thus, 54 initial dates are assigned for the simulation series. An example of RMM diagram is shown in Supplementary Fig. 1, marked with initial dates assigned for the two MJO cases projected on the diagram.

### Forecast skill evaluation

Forecast skills are assessed by COR between the reference and simulated RMM indices, formulated as


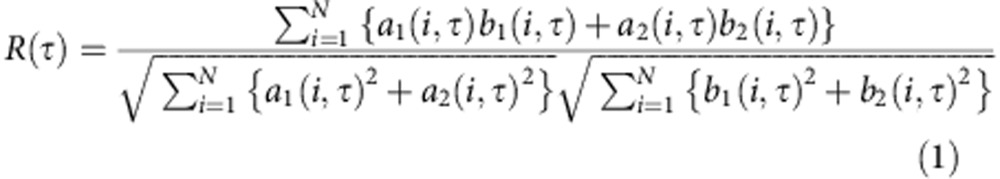


where *R, i*, *N*, *τ*, *a* and *b* are the COR score, labels for each simulation, total number of simulations, lead time from the initial date, RMM indices of the reference data and RMM indices of the model output, respectively. Climatologies are required on obtaining RMM indices, which are unavailable for our model. We use climatologies of the ERA-Interim reanalysis data as substitutes. This may degrade the model skill if the climatologies of the model considerably differ from those of the reanalysis.

### Sample differences between NICAM and operational GCMs

The operational model skills indicated in Fig. 1 are for late 2008–2012, and include simulations initiated nearly every day during November–March. The operational models apply ensemble forecasts, that is, the skills are calculated for an average of multiple simulations initiated at the same day with perturbed initial conditions. NICAM skills are those for the 54 initial dates that belong to phases assigned by the procedure described above. All of the initial dates belong to phase 8, 1 or 2, and are between October and March of 2003–2012. The mean initial amplitude of the MJO is 1.48. Ensemble forecast is not applied.

## Additional information

**How to cite this article:** Miyakawa, T. *et al.* Madden–Julian Oscillation prediction skill of a new-generation global model demonstrated using a supercomputer. *Nat. Commun.* 5:3769 doi: 10.1038/ncomms4769 (2014).

## Supplementary Material

Supplementary Information Supplementary Figures 1-11
