# Supplementary material for: Madden–Julian Oscillation prediction skill of a new-generation global model demonstrated using a supercomputer
Source: Nat Commun. 2014 May 6;5:3769. doi: 10.1038/ncomms4769 (PMC4024761; doi:10.1038/ncomms4769)
Supplement: Supplementary Information — Supplementary Figures 1-11 [file ncomms4769-s1.pdf]

Supplementary information

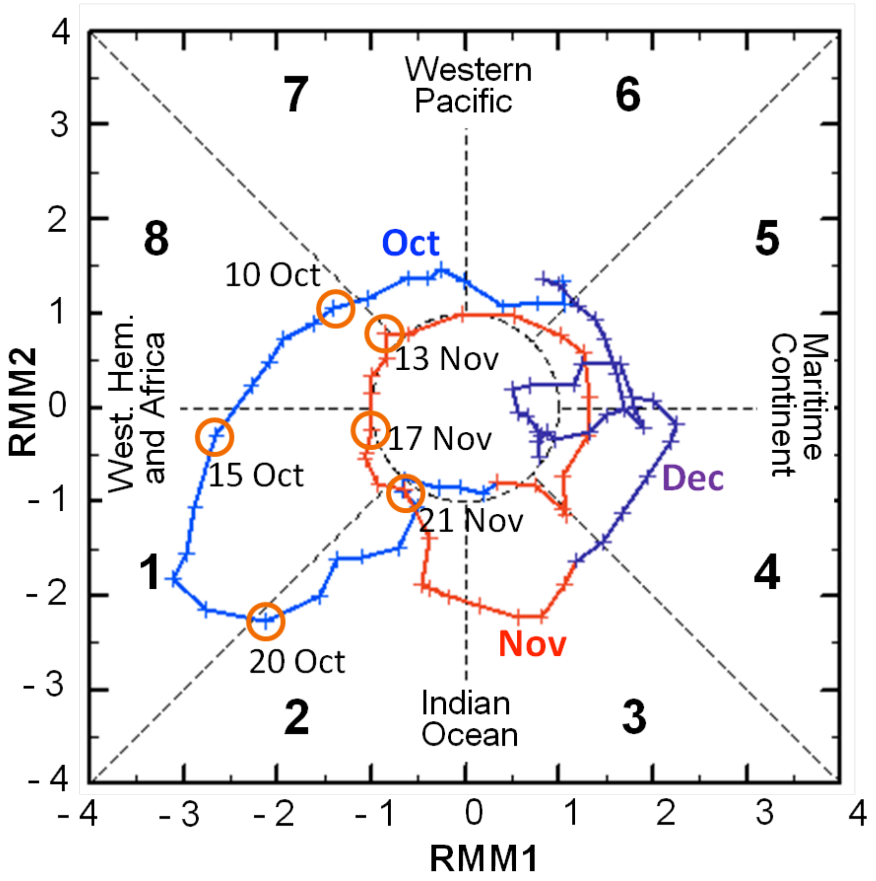

Supplementary Figure 1. RMM indices plotted through 1 Oct to 31 Dec 2011

Blue, red, and purple correspond to Oct, Nov, and Dec respectively. Phases are numbered (1 - 8) on the figure. Two MJO cases are identified during this period, i.e., the plots move counterclockwise through phase 2 to phase 5 twice. The orange circles mark the initial dates assigned for the simulations, i.e., the first dates that the RMM plots fall in phases 8, 1, and 2 in each of the identified MJO cases. The RMM indices are provided by the Bureau of Meteorology (http://www.bom.gov.au/climate/mjo/).

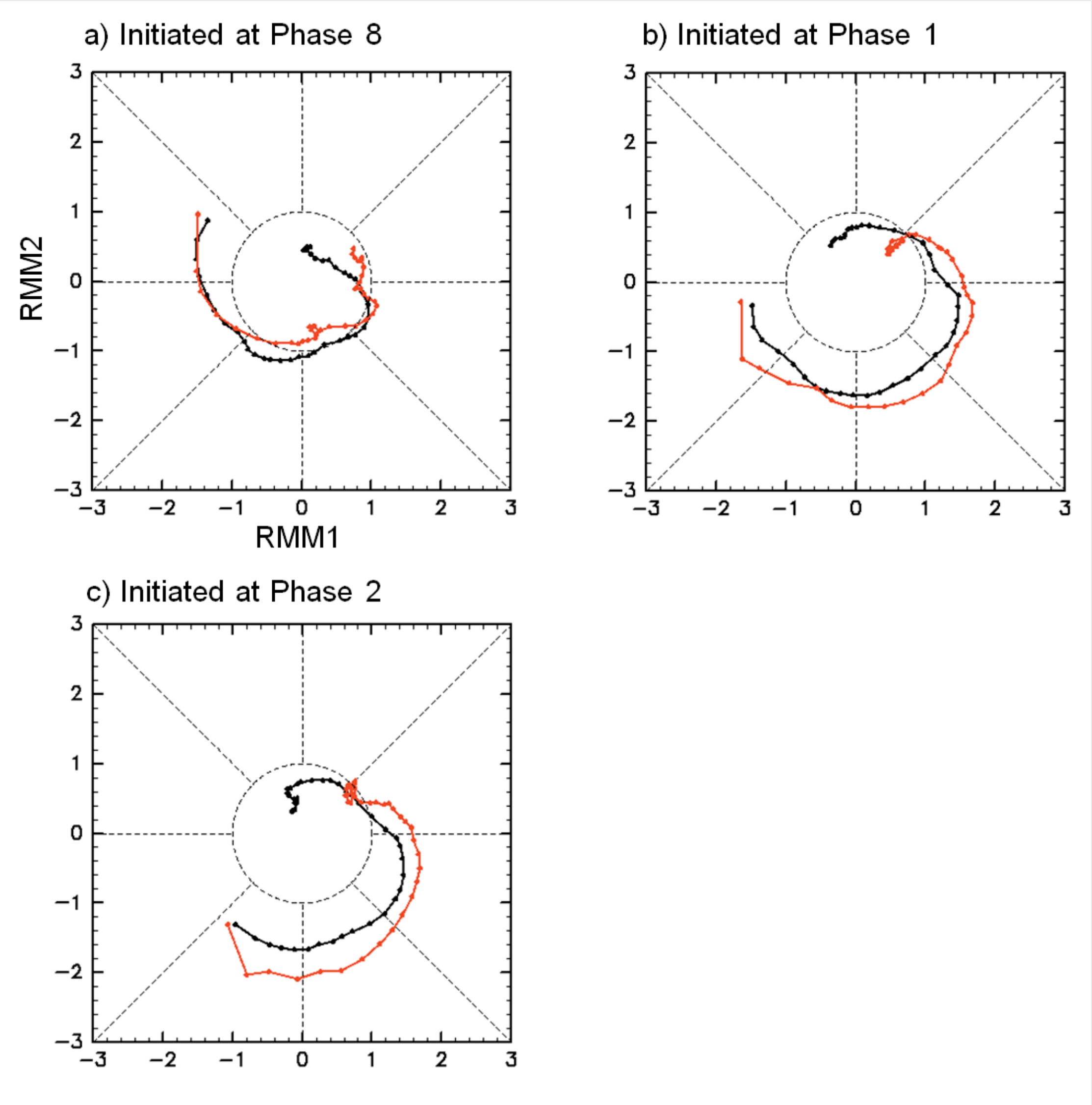

Supplementary Figure 2. Composited RMM indices for observation and simulation

a) Observation (black), and b) simulation (red). Composites consist of 17 cases (initiated at phase8), 18 cases (phase1), and 19 cases (phase2) respectively.

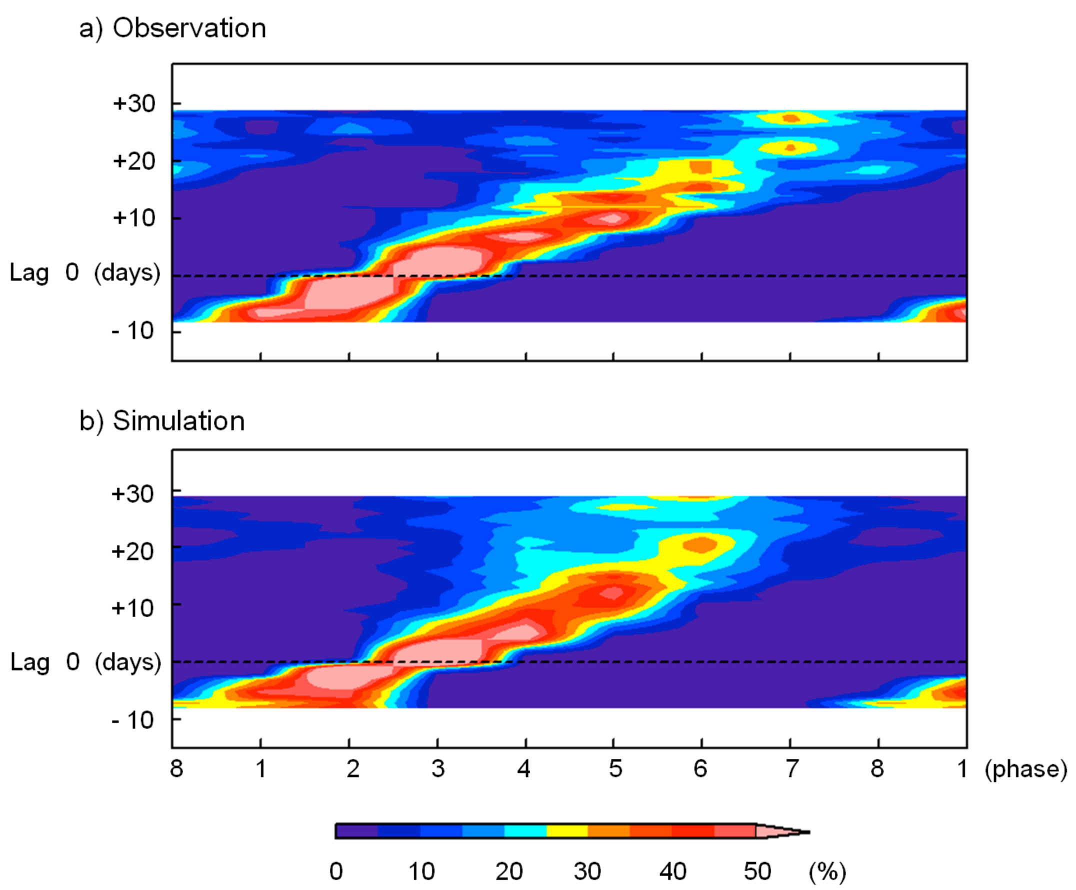

Supplementary Figure 3. Lagged composite of MJO phase probability distribution

Lag 0 is set to the 1st day the 5-day running averaged RMM index falls in Phase 3. Colours are drawn at lag-days where the composite consists of more than 30 samples for both observation and simulation.

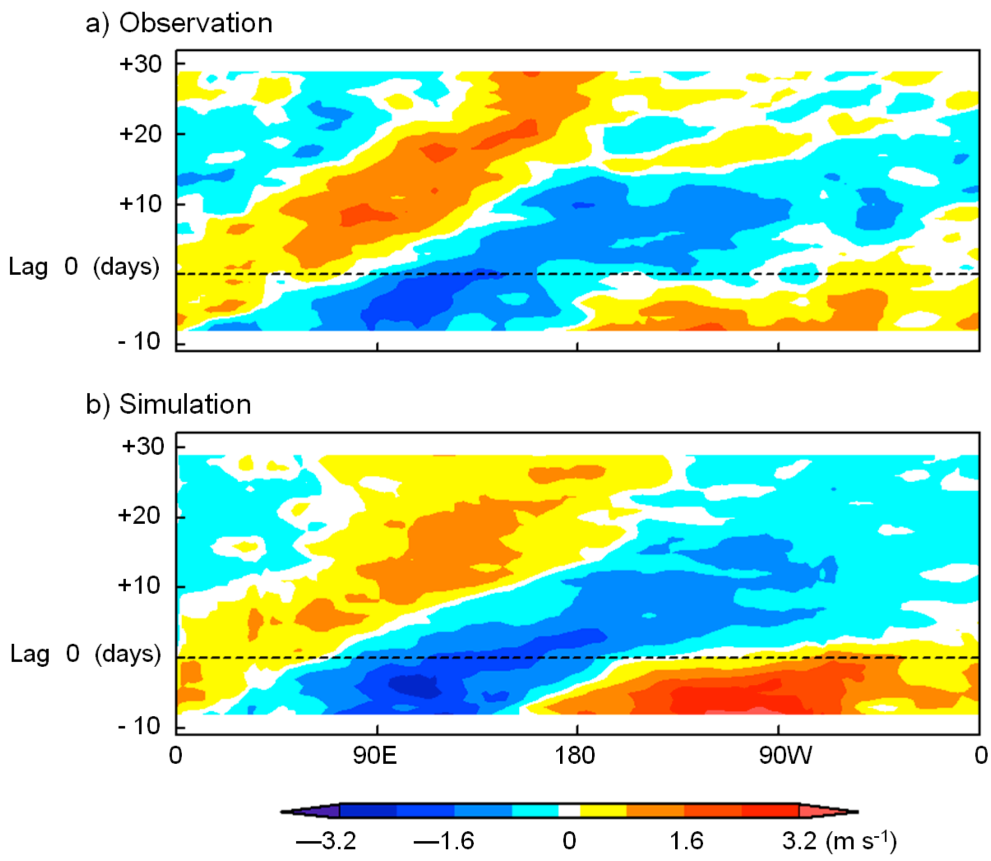

Supplementary Figure 4. Lagged composite of 850 hPa zonal wind anomalies

Lag 0 is set to the 1st day the 5-day running averaged RMM index falls in Phase 3. Anomalies are calculated as deviations from 40-day mean values of each case, and averaged over 20S - 20N. Colours are drawn at lag-days where the composite consists of more than 30 samples for both observation (ERA-interim) and simulation.

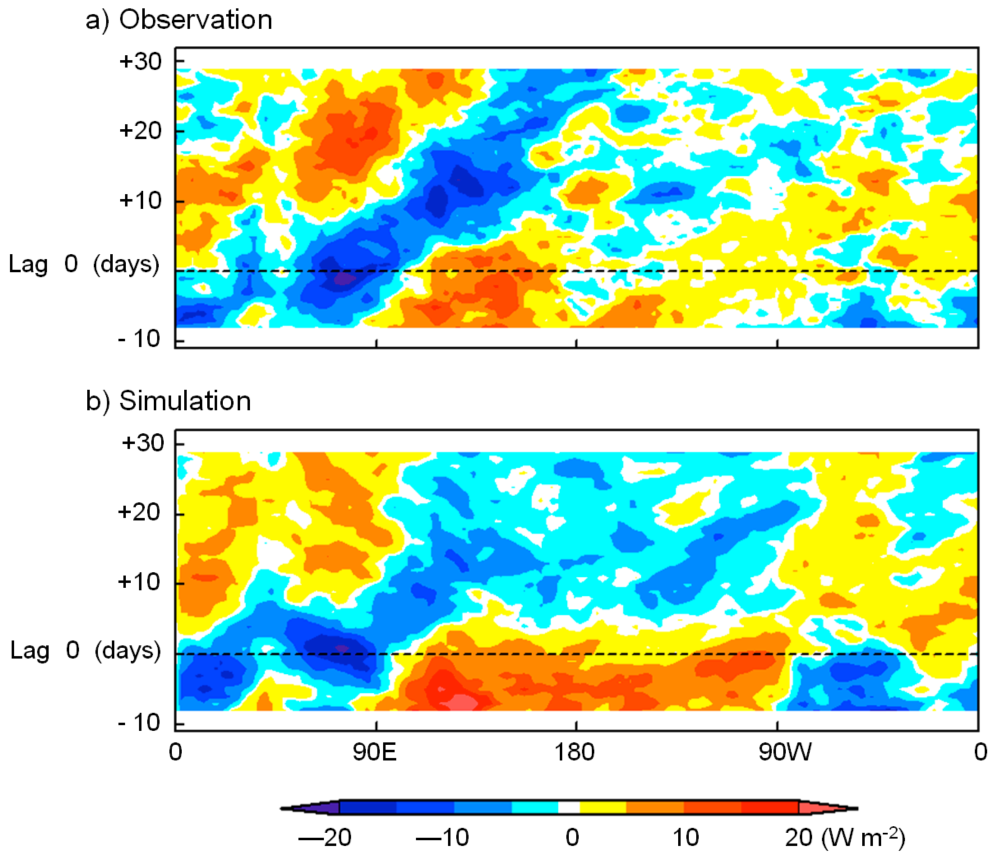

Supplementary Figure 5. Lagged composite of OLR anomalies

Lag 0 is set to the 1st day the 5-day running averaged RMM index falls in Phase 3. Anomalies are calculated as deviations from 40-day mean values of each case, and averaged over 20S - 20N. Colours are drawn at lag-days where the composite consists of more than 30 samples for both observation (NOAA-OLR) and simulation.

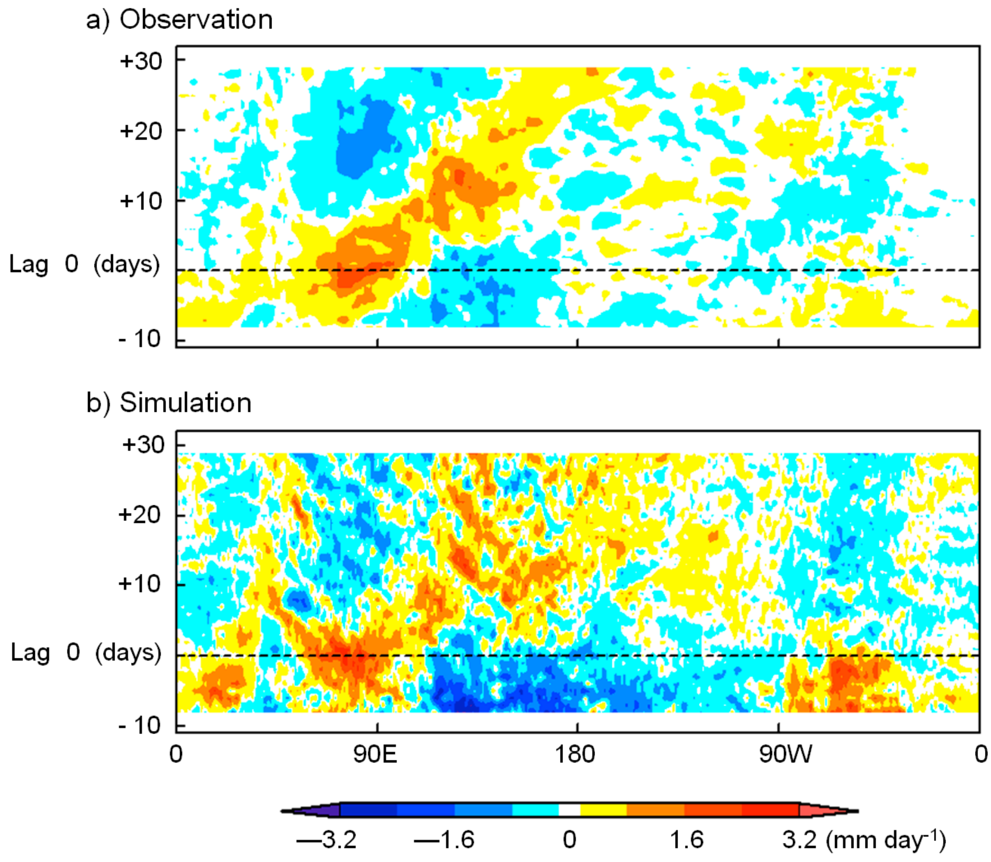

Supplementary Figure 6. Lagged composite of precipitation anomalies

Lag 0 is set to the 1st day the 5-day running averaged RMM index falls in Phase 3. Anomalies are calculated as deviations from 40-day mean values of each case, and averaged over 20S - 20N. Colours are drawn at lag-days where the composite consists of more than 30 samples for both observation (GPCP) and simulation.

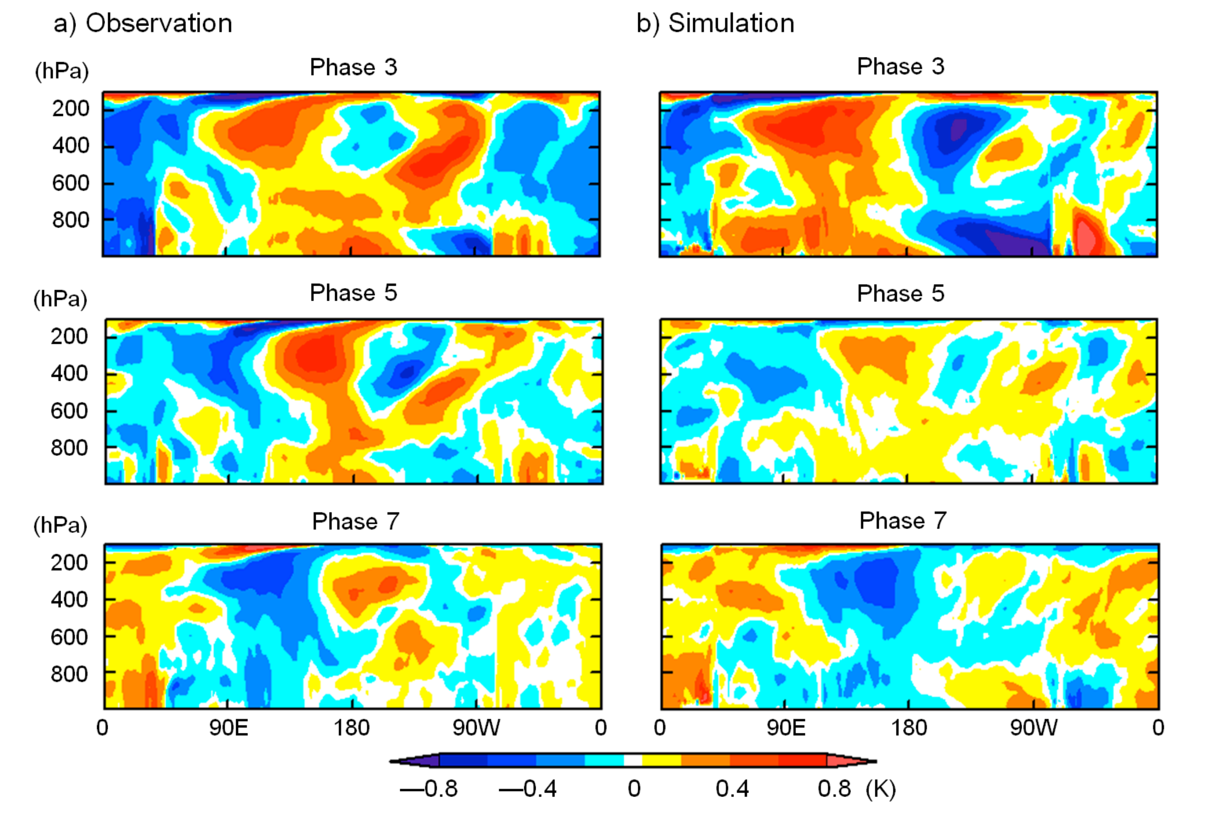

Supplementary Figure 7. Temperature anomaly composites for different MJO phases

Zonal-height sections of a) ERA-interim, and b) NICAM. Anomalies are calculated as deviations from 40-day mean values of each case, and averaged over 8S - 8N.

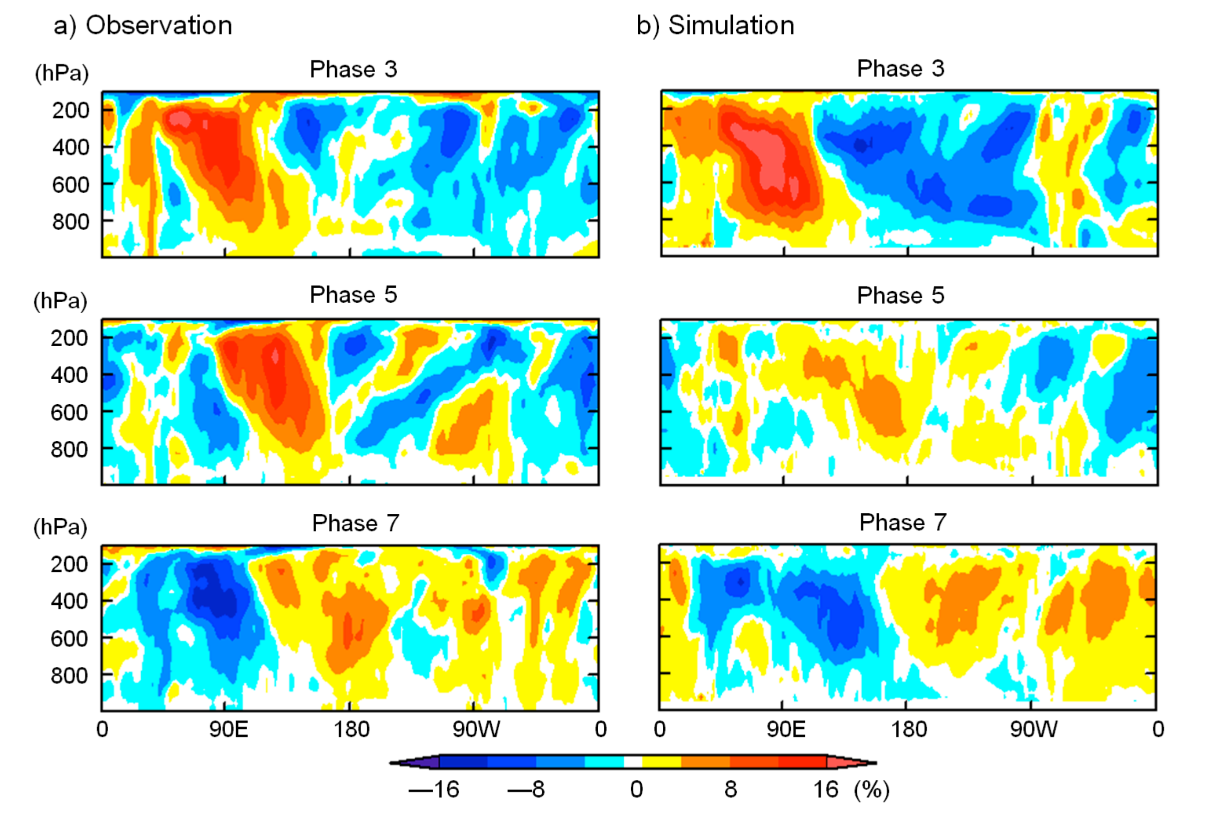

Supplementary Figure 8. Relative humidity anomaly composites for different MJO phases

Zonal-height sections of a) ERA-interim, and b) NICAM. Anomalies are calculated as deviations from 40-day mean values of each case, and averaged over 8S - 8N.

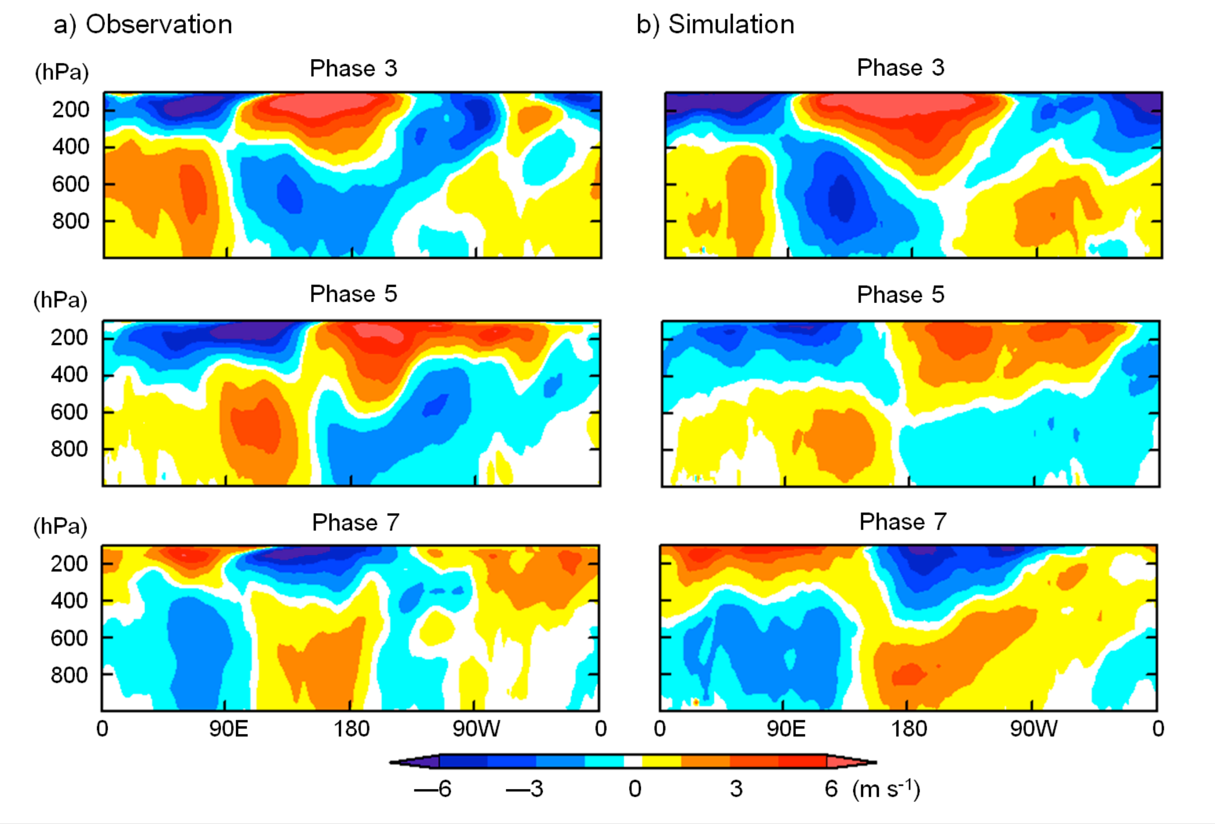

Supplementary Figure 9. Zonal wind anomaly composites for different MJO phases

Zonal-height sections of a) ERA-interim, and b) NICAM. Anomalies are calculated as deviations from 40-day mean values of each case, and averaged over 8S - 8N.

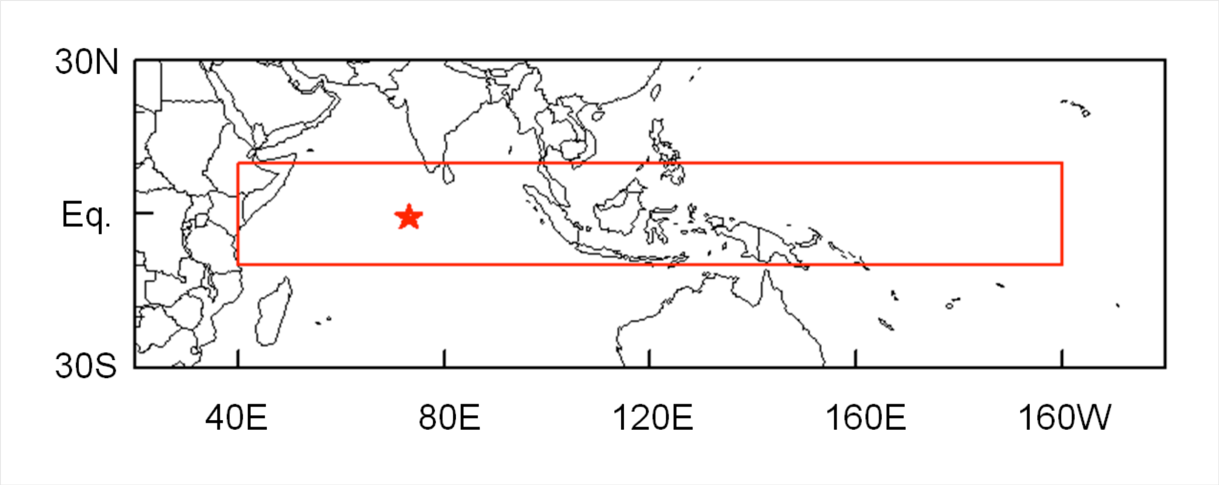

Supplementary Figure 10. The region used for the two-daily series of cloud and rain

The red rectangular area (10S - 10N, 40E - 160W) indicates the region used for the two-daily series of cloud and rain (Fig. 3 of the main paper). The sounding site on the Gan Island (73.2E, 0.7S) is marked on the map.

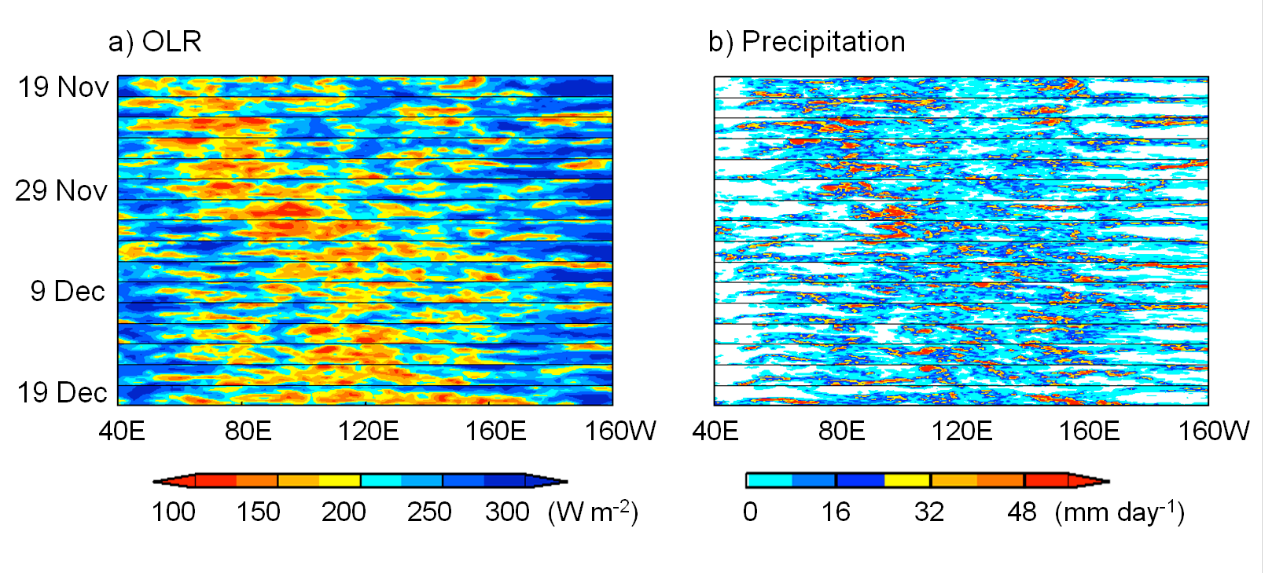

Supplementary Figure 11. Two-daily series of cloud and rain by a 7-km mesh simulation

a) OLR by a simulation that apply 7-km mesh and initialized at 00 UTC 17 Nov, 2011. Smaller values correspond to higher cloud tops. b) Precipitation by the simulation. The figures consist of slices that show horizontal snapshots of the tropical Indian to the western Pacific Ocean (10S - 10N, 40E - 160W; indicated in Supplementary Fig. 10). The resolutions of the OLR and precipitation are respectively lowered to 2.5-degree mesh and 1-degree mesh, as done in Fig. 3 of the main paper.
